# Does Lower Psychological Need Satisfaction Foster Conspiracy Belief? Longitudinal Effects Over 3 Years in New Zealand

**DOI:** 10.1177/01461672241292841

**Published:** 2024-12-13

**Authors:** Elianne A. Albath, Rainer Greifeneder, Karen M. Douglas, Aleksandra Cichocka, Mathew D. Marques, Marc S. Wilson, John R. Kerr, Chris G. Sibley, Danny Osborne

**Affiliations:** 1 University of Basel, Switzerland; 2 University of Kent, UK; 3 La Trobe University, Melbourne, Victoria, Australia; 4 Victoria University of Wellington, New Zealand; 5 University of Otago, Wellington, New Zealand; 6 The University of Auckland, New Zealand

**Keywords:** conspiracy belief, psychological need satisfaction, longitudinal analysis, random-intercept cross-lagged panel model

## Abstract

Although conspiracy belief may arise from a frustration of psychological needs, research has yet to investigate these relationships over time. Using four annual waves of longitudinal panel data in New Zealand (2019–2022; *N* = 55,269), we examined the relationship between four psychological needs (namely belonging, control, meaning in life, and self-esteem) and conspiracy belief. Results from four random-intercept cross-lagged panel models reveal stable between-person effects indicating that those whose core needs are less satisfied tend to exhibit higher levels of conspiracy belief across time. Within-person analyses further identify small cross-lagged effects within individuals: *decreases* in levels of control and belonging, as well as *increases* in levels of meaning in life, temporally precede increases in conspiracy belief. Within-person fluctuations in conspiracy belief also have negative cross-lagged associations with control (but not with the three other needs). These data provide novel insights into the psychological factors that foster conspiracy belief.

Despite their varied manifestations, conspiracy theories share the belief that a group of individuals (or entities) have secretly coordinated to achieve a malevolent outcome, and that their activities are of public significance but not widely known (Douglas & Sutton, 2023). Associations with conspiracy belief are often detrimental and include voter abstention (Jolley & Douglas, 2014), the tendency to ignore health-promoting recommendations (Coelho et al., 2022; see also van Mulukom et al., 2022), and the proclivity to use violence to express discontent (Rottweiler & Gill, 2022). To date, the theorized psychological antecedents of conspiracy belief are myriad (Douglas & Sutton, 2023) and include psychological motivations (Douglas et al., 2017), personality characteristics (e.g., maladaptive personality facets; Stasielowicz, 2022; Swami et al., 2016), and relatively stable individual needs (e.g., need for uniqueness; Imhoff & Lamberty, 2017; Lantian et al., 2017). Situational variables such as societal change that spark feelings of uncertainty may also elicit conspiracy belief (van Prooijen & Douglas, 2017; cf. Uscinski, Enders, Klofstad & Stoler, 2022).

To date, much of the research examining these relationships has utilized cross-sectional data. Yet understanding how psychological needs longitudinally affect conspiracy belief may be particularly informative. For instance, being socially excluded and ignored by others, an experience that affects the basic needs of belonging, control, self-esteem, and meaning (Williams, 2009) correlates positively with conspiracy belief (Poon et al., 2020) and openness to extreme groups (Hales & Williams, 2018), as well as support for terrorist groups and the use of violence on their behalf (Pfundmair, 2019). Unfortunately, robust evidence on the longitudinal associations between need satisfaction and conspiracy belief is scarce (Liekefett et al., 2023; Uscinski et al., 2022).

The present research addresses this limitation and aims to answer two questions: (a) Does lower psychological need satisfaction predict increases in conspiracy belief over time? and (b) Do increases in conspiracy belief predict greater need satisfaction over time? To answer these questions, we chose an analytical approach that separates the stable, trait-like differences that exist between people across time from the time-specific, within-person departures from these general dispositions (namely, a Random Intercept Cross-lagged Panel Model; RI-CLPM; Hamaker et al., 2015). In doing so, we increase understanding of these two potential longitudinal pathways by investigating the temporal ordering of within-person changes in psychological need satisfaction and conspiracy belief. Such insights can help inform whether psychological needs precede conspiracy belief, or conspiracy belief precedes psychological needs.

## Psychological Needs and Conspiracy Belief

Conspiracy theories seek to explain significant social and political events by positing the existence of covert plots masterminded by two or more (typically powerful) actors (Douglas et al., 2019). Given that belief in one conspiracy theory correlates positively with believing in another (Goertzel, 1994), people may have a *tendency* to believe that malevolent groups conspire, as well as a predisposition to distrust official accounts of impactful events (Bruder et al., 2013). Here, we use the term *conspiracy belief* to describe both belief in specific conspiracy theories (e.g., “The U.N. is trying to take control of the United States”; Abalakina-Paap et al., 1999) and belief in more general notions of conspiracy (e.g., “I think that the official version of the events given by the authorities very often hides the truth”; Lantian et al., 2016).

Recent research on conspiracy belief demonstrates that, while belief in conspiracy theories shows some degree of intrapersonal variability, it is generally stable over time. For example, Williams et al. (2024) found that people’s conspiracy belief assessed across seven monthly surveys had an intraclass correlation coefficient (ICC) of 0.91, indicating considerable within-person stability over time. This pattern is consistent with findings from other contexts, where within-person variability in conspiracy belief remains minimal despite the use of different measures. For instance, Jolley et al. (2022) observed a strong correlation (*r* = .78) between Brexit conspiracy belief across two time points 9 days apart on either side of the referendum vote. Similarly, Liekefett et al. (2023) reported little within-person variation in conspiracy belief across four assessments (ICC in Study 1 = 0.80 and ICC in Study 2 = 0.75). These studies collectively demonstrate high levels of stability in conspiracy belief, while also identifying some underlying dynamism through small, yet discernible, fluctuations.

Conspiracy belief may stem, at least in part, from a desire to restore one’s understanding and clarity about the world (epistemic motive), offer a feeling of control and security (existential motive), and increase individual, as well as collective, self-esteem (social motive; Douglas et al., 2017). The notion that individuals strive to increase basic psychological need satisfaction corroborates separate literature. For instance, being socially ignored and excluded by others, a hurtful and threatening experience with severe negative downstream consequences (Reinhard et al., 2020) threatens four basic needs: control, belonging, self-esteem, and meaning in life (Williams, 2009). Importantly, these four basic psychological needs overlap with the motives thought to foster conspiracy belief. For instance, the need for control corresponds to the existential motive (i.e., feeling safe and secure), while feeling good about oneself (need for self-esteem) and close to others (need to belong) overlaps with the social motive. While some of these needs overlap with different motivations (e.g., need for control reflects both epistemic and existential motives), they share the general idea that lower psychological need satisfaction correlates positively with conspiracy belief. Accordingly, experiences of social exclusion are associated with stronger conspiracy belief (Poon et al., 2020). In the following sections, we provide a brief overview of how each of these needs correlates with conspiracy belief. Although all these needs are closely connected, they may also have their own, unique associations with conspiracy belief (Bowes et al., 2023; Dow et al., 2022).

### Need for Control

The existential motive underlying conspiracy belief describes individuals’ need to feel safe, secure, and in control of their environment (Douglas et al., 2017). Consistent with compensatory control theory (Kay et al., 2009), threats to one’s need for personal control motivate individuals to restore a sense of control through compensatory strategies. For instance, individuals exaggerate the influence of enemies to compensate for reduced control (Sullivan et al., 2010). Similarly, belief in conspiracy theories may help people restore a sense of personal control. The current evidence for the impact of perceived control and power on conspiracy belief is, however, mixed. On one hand, correlational evidence suggests that lack of control and power are associated with stronger conspiracy belief (Abalakina-Paap et al., 1999; Jolley & Douglas, 2014; Stojanov et al., 2020; van Prooijen & Acker, 2015). On the other hand, a recent meta-analysis found limited evidence for a causal effect between reduced levels of control and conspiracy belief (Stojanov & Halberstadt, 2020). Specifically, experimental manipulations of control had a small effect on specific conspiracy beliefs, but no effect on measures that capture belief in conspiracy theories more generally (Stojanov & Halberstadt, 2020).

In sum, perceptions of control correlate negatively with conspiracy belief, with some experimental evidence showing that a temporary loss of control increases conspiracy belief. What is less known, however, is whether lower levels of perceived control outside the laboratory precede increases in conspiracy belief. Moreover, it is currently unknown whether conspiracy belief *increases* feelings of control. Research exploring related constructs indicates that conspiracy belief is, if anything, linked to an increase in anxiety and a heightened sense of existential threat over time (Liekefett et al., 2023). Accordingly, this association may also extend to feelings of control.

### Need to Belong

A sense of belonging is essential to individuals’ well-being and is a powerful motivator of their behaviors (Baumeister & Leary, 1995). As such, an unfulfilled need to belong can fuel the desire to restore individuals’ sense of belonging (Biddlestone et al., 2021). For instance, individuals who experience social exclusion more frequently or who are excluded in experimental studies report stronger conspiracy belief (Poon et al., 2020). Conversely, self-affirmations can eliminate the effect of social exclusion on conspiracy belief, suggesting that conspiracy belief helps people cope with this threat (Poon et al., 2020). Importantly, social *inclusion* can increase conspiracy belief among people whose self-esteem is unstable (van Prooijen, 2016).

At a societal level, low feelings of belonging can become chronic when individuals belong to disadvantaged and minority groups (Biddlestone et al., 2021). These findings extend to groups whose political views are not represented in government. Indeed, those who hold more extreme political opinions also report stronger conspiracy belief (Imhoff et al., 2022). In sum, people who experience acute or chronically low levels of belonging show stronger conspiracy belief, but research has yet to determine the direction of causation.

### Need for Self-Esteem

General self-esteem (i.e., having a positive attitude about oneself) is a core component of psychological well-being (Rosenberg et al., 1995) and is closely linked to social belongingness (Leary & Baumeister, 2000). For instance, a 12-year longitudinal study found that self-reported self-esteem significantly predicted life-span trajectories of relationship satisfaction, job satisfaction, positive and negative affect, and depression (Orth et al., 2012). Accordingly, people endorse conspiracy theories in an attempt to feel good about themselves and the groups to which they belong (Douglas et al., 2017). Indeed, early theorizing proposed that low self-esteem is associated with stronger conspiracy belief (Abalakina-Paap et al., 1999). Newer findings, however, suggest that low self-esteem is only weakly associated with conspiracy belief after accounting for narcissism (Cichocka et al., 2016)—results corroborated by a recent meta-analysis (Stasielowicz, 2022). However, given that many studies rely on cross-sectional assessments, it is unclear whether fluctuations in self-esteem temporally precede deviations in conspiracy belief (or vice versa). Accordingly, the between-person predictive value of self-esteem on conspiracy belief may be small, but temporary within-person deviations in self-esteem may still predict increases in conspiracy belief over time (or vice versa).

### Need for Meaning in Life

Perceiving one’s life to be filled with purpose is strongly associated with positive affect and psychological well-being and is an essential aspect of laypeople’s assumptions of what makes a “good life” (King et al., 2006). Indeed, when describing meaningful life events, individuals typically report events that involve interpersonal relationships or self-improvement (King et al., 2006). The Meaning Maintenance Model further suggests that individuals pursue self-esteem because it provides them meaning (Heine et al., 2006). For instance, belonging to a highly regarded group provides individuals with a sense of purpose (e.g., by achieving a goal beneficial to the group). As such, meaning in life is closely related to other social needs such as self-esteem and belongingness (Lambert et al., 2013). And like other needs, when individuals lack a sense of meaning, they initiate compensatory processes to restore this need (Heine et al., 2006; Steger et al., 2008; Williams, 2009). For instance, after a severe earthquake in New Zealand, those affected by the earthquake were more likely to believe in God compared with those living in different parts of the country (Sibley & Bulbulia, 2012). Thus, people may turn to conspiracy theories when their need for meaning in life is frustrated (Schöpfer et al., 2023). Indeed, belief in conspiracy theories seems to allow individuals to attain or maintain a sense of meaning (Newheiser et al., 2011; Schöpfer et al., 2023).

## Overview of this Study

So far, we have argued that deviations in psychological need satisfaction may precede conspiracy belief, or conversely, that deviations in conspiracy belief may precede fluctuations in psychological need satisfaction. Although the latter direction has received less empirical attention, current longitudinal evidence suggests that conspiracy belief does not have beneficial consequences (Liekefett et al., 2023). To understand more about these relationships, we use four annual waves of longitudinal panel data from a nationwide random sample of adults in New Zealand to investigate whether the temporal associations between four core psychological needs (control, belonging, self-esteem, and meaning in life) and conspiracy belief are bidirectional. For instance, we examine whether deviations in meaning in life precede deviations in conspiracy belief one year later and whether deviations in conspiracy belief are likewise associated with future deviations in the perceived sense of purpose in life.

To investigate these associations longitudinally, we conduct four separate RI-CLPMs. Whereas traditional CLPMs confound stable, between-person differences that persist over time with within-person fluctuations, RI-CLPMs estimate a random intercept to account for stable between-person differences (Hamaker et al., 2015; Osborne & Little, 2024). The resultant autoregressive and cross-lagged effects, therefore, reflect residual within-person deviations from one’s trait-level means. Accordingly, RI-CLPMs are quickly becoming the method of choice for examining within-person associations that form the core of psychological theory (Hamaker, 2023).

## Method

### Participants

We used data from the New Zealand Attitudes and Values Study (NZAVS), an ongoing, longitudinal panel study based on a nationwide random sample of New Zealand adults. The NZAVS collects data on social attitudes, personality, ideology, and health outcomes (for more information on the NZAVS and its measures, see https://osf.io/75snb/). Our measure of conspiracy belief has been part of the annual assessments in 2019 (Time 11), 2020 (Time 12), 2021 (Time 13), and 2022 (Time 14). Our focal sample includes 55,269 respondents; the number of respondents in each analysis varies due to missing data but is specified when reporting the results. *Table 1* provides an overview of key demographics at each assessment occasion.

**Table 1.** Sample Demographic Characteristics Over Four Annual Waves.

Characteristic	Time 11 (October 2019–September 2020)	Time 12 (October 2020–September 2021)	Time 13 (October 2021–September 2022)	Time 14 (October 2022–September 2023)
Age (Mean [*SD*])	52.05 (13.86)^a^	53.45 (13.69)^b^	54.89 (13.66)^c^	54.11 (15.70)^d^
Gender (Female; *n* [%])	27,149 (64.07)	24,499 (63.99)	21,768 (64.26)	21,234 (63.67)
Ethnicity (*n*)				
NZ European/Pākehā	35,117	32,310	28,923	28,148
Māori	4,314	3,374	3,047	3,099
Pacific	819	708	492	480
Asian	1,714	1,443	1,147	1,181

*Note.* Demographic information is based on the number of participants who answered the respective questions.

a *n* = 42,681. ^b^ *n* = 38,550. ^c^ *n* = 34,131. ^d^ *n* = 33,721.

### Measures

Except for control, all measures were assessed at each time point (Time 11, Time 12, Time 13, and Time 14) using the same wording and with the same 7-point scale from 1 (*strongly disagree*) to 7 (*strongly agree*). Control was assessed at three time points (Times 11–13). When multiple items were used to measure the same construct, we averaged these items at each wave to create an index.

**Conspiracy belief** was assessed with a single item adapted from Lantian et al. (2016): “I think that the official version of major world events given by authorities often hides the truth.” The original item contains a preamble with examples of debated conspiracy theories (e.g., the assassination of JFK), in addition to an explanation that official versions of these events could have been given by powerful groups to hide the truth from the public. In the NZAVS, the item was assessed without this preamble to keep the length of the questionnaire acceptable. Lantian et al. (2016) found that this single item displayed acceptable (a) convergent validity with other validated measures of belief in conspiracy theories (e.g., paranormal belief and interpersonal trust), (b) discriminant validity (e.g., self-consciousness), and (c) test–retest reliability.

**Control** was assessed using these two items: “I do not have enough power or control over important parts of my life” and “Other people have too much power or control over important parts of my life.” These reverse-scored items had a medium-to-strong positive correlation with each other at each measurement occasion: Time 11 (Spearman’s rho, *ρ* = .55, *p* < .001), Time 12 (*ρ* = .50, *p* < .001), and Time 13 (*ρ* = .54, *p* < .001).

**Felt belongingness** was assessed using these three items from Hagerty and Patusky (1995): “I. . .” “know that people in my life accept and value me,” “feel like an outsider” [reverse-scored], and “know that people around me share my attitudes and beliefs.” The items had low reliability at each measurement occasion: Time 11 (α = .60), Time 12 (α = .64), Time 13 (α = .60), and Time 14 (α = .59).^1^

**Self-esteem** was assessed using these three items from Rosenberg (1965): “On the whole am satisfied with myself,” “Take a positive attitude toward myself,” and “Am inclined to feel that I am a failure” (reverse-scored). The items showed high reliability at each measurement occasion: Time 11 (α = .82), Time 12 (α = .83), Time 13 (α = .82), and Time 14 (α = .82).

**Meaning in life** was assessed using these two items from Steger et al. (2006): “My life has a clear sense of purpose” and “I have a good sense of what makes my life meaningful.” The items correlated strongly at each measurement occasion: Time 11 (*ρ* =.60, *p* < .001), Time 12 (*ρ* = .61, *p* < .001), Time 13 (*ρ* = .61, *p* < .001), and Time 14 (*ρ* = .62, *p* < .001).

### Statistical Analyses

First, we examined the measurement invariance of our two three-item measures (i.e., self-esteem and felt belongingness) across all four assessment occasions. Evidence of noninvariance would imply that the measurement of a construct changes across assessment occasions and, thus, is a threat to construct validity. Accordingly, demonstrating longitudinal measurement invariance helps increase confidence that the indicators of a latent variable capture the underlying construct in similar ways across time and that a given latent variable is on a similar metric at each assessment occasion (see Widaman et al., 2010). Thus, measurement invariance ensures that we are comparing “apples to apples” when investigating latent variables assessed at multiple time points.

Second, to test whether temporary declines from one’s trait-level psychological need satisfaction precede conspiracy belief, we estimated four separate RI-CLPMs using the lavaan package (Rosseel, 2012) in R (R Core Team, 2022). Specifically, we separately estimated RI-CLPMs in which one of the four psychological needs (i.e., control, belonging, self-esteem, and meaning in life) and conspiracy belief at Time 11, Time 12, and Time 13 were used to predict each other at Time 12, Time 13, and Time 14, respectively. Although two of our models included a measure of psychological need that could theoretically be estimated as a latent variable at each assessment occasion (i.e., self-esteem and felt belongingness were both assessed with three items), we encountered convergence issues when attempting to estimate multiple-indicator RI-CLPMs. As such, we followed the standard approach to RI-CLPMs and used an index (i.e., the items averaged together) of each construct at each assessment occasion when estimating each of the four RI-CLPMs. These measured variables at each assessment were then partitioned into between-person latent variables (i.e., the random intercepts) and single indicator latent variables at each assessment occasion to capture the time-specific, within-person deviations from participants’ trait-level means. Error variances for these indicator variables were thus assumed to be fully reliable, given these standard estimation assumptions.

Because we had no theoretical reasons to predict the strength of these associations would vary over time, we modeled these associations as a stationary process. That is, the autoregressive and cross-lagged associations from Time 11 to Time 12 were constrained to be equal to their respective associations from Time 12 to Time 13, as well as from Time 13 to Time 14. The RI-CLPMs also estimated random intercepts for the four psychological needs and conspiracy belief. These random intercepts reflect participants’ average levels of need satisfaction and conspiracy belief across all four assessments. This was done by separately fixing the factor loadings of each index variable at each measurement occasion to 1 and by allowing the two respective random intercepts to correlate. In supplementary analyses, we also estimated a comprehensive RI-CLPM including all five constructs (four needs and conspiracy belief) across Times 11–13 (note: We estimated a comprehensive model with three [instead of four] annual assessments because control was only assessed at Times 11–13). The results of this analysis can be found on the project’s Open Science Framework (OSF) webpage (https://osf.io/h9zfq/).

We evaluate model fit using the comparative fit index (*CFI*), root mean square error of approximation (*RMSEA*), and standardized root mean squared residual (*SRMR*; Hu & Bentler, 1999). The tests of measurement invariance and RI-CLPMs were estimated using full information maximum likelihood (FIML) to address missing data. Missing data are inevitable in longitudinal research, and FIML offers two advantages. First, unlike other methods, FIML does not rely on imputing missing values or the assumption that data are missing completely at random (Enders, 2001). Second, FIML efficiently utilizes all available data without using list-wise or case-wise deletion—two alternative approaches to missing data that often lead to a loss of information. In comparison with list-wise or case-wise deletion, FIML demonstrates superior performance in generating unbiased and efficient parameter estimates while effectively managing Type 1 error rates (Enders & Bandalos, 2001). Unless noted, we report unstandardized estimates throughout the article.

Finally, a note on effect sizes in RI-CLPMs: Given that cross-lagged effects represent predictions over time, they are typically substantially smaller than cross-sectional associations of the same constructs. This is because RI-CLPMs control for autoregressive effects and correlations at the previous time point, as well as the stable, between-person differences that persist over time. Accordingly, a recent meta-analysis of the CLPM (and RI-CLPM) literature suggests treating (standardized) effect sizes of .03, .07, and .12 as small, medium, and large, respectively (Orth et al., 2022). Although these benchmarks should not be used to determine whether an effect is relevant or not, they help indicate whether an effect is smaller/larger than other cross-lagged effects in psychology. As such, we also report (where appropriate) standardized estimates (β) for cross-lagged effects to aid interpretation.

## Results

### Measurement Invariance

Longitudinal measurement invariance involves sequentially estimating increasingly restrictive measurement models, beginning with a configural invariant model in which the factor loading patterns for a construct are identical at each assessment occasion. As such, we estimated a measurement model in which the same three items that comprised self-esteem at Time 11 were the same three items that comprised self-esteem at Times 12–14. Likewise, the same three items that comprised felt belongingness at Time 11 were the same three items that comprised felt belongingness at Times 12–14. To identify our model, we used a fixed factor approach by fixing the latent variable means and variances to 0 and 1 (respectively) at each assessment occasion and freely estimating all three-factor loadings for each construct at each assessment (Little, 2013). We also allowed the residuals for congeneric items to correlate (e.g., the residual variance of the first indicator of self-esteem at Time 11 correlated with the residual variance of the first indicator of self-esteem at Times 12–14). Finally, we estimated our models FIML estimates in Mplus v8.10 (Muthén & Muthén, 1998–2017).

Table 2 demonstrates that our initial longitudinal measurement model with configural invariance fit these data well, ꭓ^2^_(188)_ = 12578.064, *CFI* = 0.974, *RMSEA* = 0.035, 90% confidence interval (CI) = [0.034, 0.035], *SRMR* = 0.041. As such, we estimated a more restrictive measurement model with metric invariance by constraining the congeneric factor loadings for each construct to equality across time and by freely estimating the latent variances for both self-esteem and felt belongingness from Times 12–14. Because the addition of these constraints did not significantly reduce model fit (i.e., ∆*CFI* < 0.01; see (Cheung & Rensvold, 2002), we estimated a more restrictive measurement model with scalar invariance by constraining the congeneric item intercepts for each construct to equality across assessments and freely estimating the latent variable means at Times 12–14. Although these additional constraints did not significantly reduce model fit (∆*CFI* = 0.007), the resultant psi covariance matrix was not positive definite. Scalar invariance is, however, only necessary when examining mean-level differences over time (Putnick & Bornstein, 2016). Therefore, our longitudinal measurement model displayed (at least) metric invariance, which is needed when comparing the strength of associations over time (i.e., the focal aim of our study).

**Table 2:** Fit Statistics for the Longitudinal Measurement Invariance Tests for Felt Belongingness and Self-Esteem (N = 55,162)

Invariance Level	Loglikelihood	χ^2^	*df*	RMSEA	RMSEA 90% CI	SRMR	CFI	∆CFI	LRT
1 Configural invariance	−1340559.857	12578.064^***^	188	.035	[.034, .035]	.041	.974	—	—
2 Metric invariance	−1340665.661	12789.671^***^	200	.034	[.033, .034]	.042	.974	<.001	211.608 ^***^
3 Scalar invariance^a^	−1341782.872	15024.094^***^	212	.036	[.035, .036]	.041	.969	.007	2234.422^***^

*Note.* Configural (same factor loading patterns), metric (equal congeneric factor loadings), and scalar (equal congeneric intercepts) models were estimated sequentially. LRT = Likelihood ratio test.

a The latent variable covariance matrix in the measurement model with scalar invariance was not positive definite.

*** *p* < .001.

### Main Analyses

Given the noted problems with a traditional CLPM (Hamaker et al., 2015; Osborne & Little, 2024), we estimated four separate RI-CLPMs to properly partition the between- and within-person associations between conspiracy belief and psychological needs.

#### Control

The first model tested whether control and conspiracy belief were associated longitudinally by estimating an RI-CLPM using data from Times 11–13. The model used data from 50,339 participants (with 37 patterns of missing data). Fit indices for the final RI-CLPM with full stationarity indicate excellent model fit, χ^2^_(5)_ = 349.48, *p* < .001; *CFI* = 0.994, *RMSEA* = 0.037 [0.034, 0.040], *SRMR* = 0.021.

**Figure 1.**
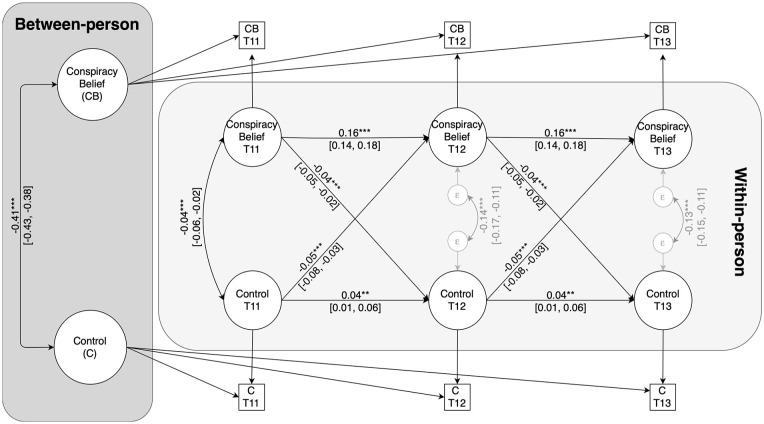
RI-CLPM of the Relationship Between Conspiracy Belief and Control. *Note*. Coefficients are unstandardized. **p < .01. ***p < .001.

At the between-person level, the random intercepts of control and conspiracy belief correlated negatively, *b* = −0.41, 95% CI = [−0.43, −0.38], *p* < .001. This indicates that individuals who generally display lower levels of control also report stronger conspiracy belief across assessments. With respect to the within-person associations, we first examined the autoregressive paths. Contrary to traditional CLPMs, autoregressive paths in RI-CLPMs do not capture the stability of a construct over time. Rather, they show whether deviations from a typical level of a construct at one wave “spill over” to deviations at the next wave (Osborne & Little, 2024). As shown in Figure 1, both autoregressive effects were positive and significant. Thus, within-person deviations from the trait levels of control and conspiracy belief at one wave carried over to within-person deviations in the same variables at the next assessment occasion.

With respect to the within-person cross-lagged relationships between conspiracy belief and control, within-person deviations from participants’ trait-level mean of control at a given assessment predicted subsequent within-person decreases in conspiracy belief, *b* = −0.05, 95% CI = [−0.08, −0.03], β = −0.04, *p* < .001. Similarly, within-person deviations from participants’ trait-level conspiracy belief at a given assessment predicted subsequent within-person decreases in control over time, *b* = −0.04, 95% CI = [−0.05, −0.02], β = −0.05, *p* < .001. The corresponding standardized regression coefficients indicate that these are small cross-lagged effects in both directions.

#### Belonging

The second model tested whether belonging and conspiracy belief were associated longitudinally by estimating an RI-CLPM using data from Times 11–14. The model used data from 55,253 participants (with 149 patterns of missing data). Fit indices for the final RI-CLPM with full stationarity indicate excellent model fit, χ^2^_(17)_ = 751.99, *p* < .001; *CFI* = 0.993, *RMSEA* = 0.028 [0.026, 0.030], *SRMR* = 0.027.

**Figure 2.**
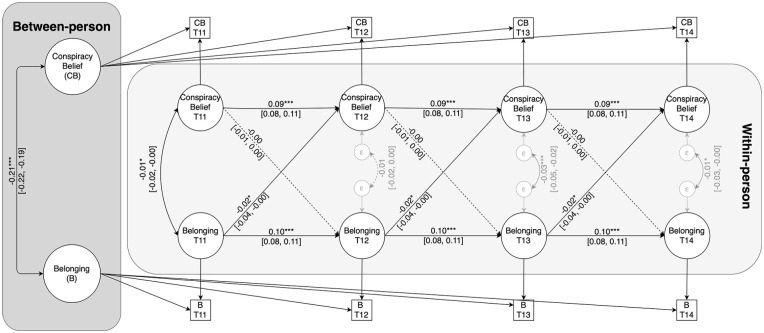
RI-CLPM of the Relationship Between Conspiracy Belief and Belonging. *Note*. Coefficients are unstandardized. Dashed lines represent nonsignificant effects. *p < .05. ***p < .001.

At the between-person level, the random intercepts of belonging and conspiracy belief correlated negatively, *b* = −0.21, 95% CI = [−0.22, −0.19], *p* < .001. Thus, individuals who generally display lower levels of belonging also report stronger conspiracy belief across assessments. With respect to within-person associations, both autoregressive effects were positive and significant, indicating that within-person deviations from the trait-level mean of the given construct at one wave carried over to within-person deviations in the same variable at the next assessment (see Figure 2). Furthermore, within-person deviations from participants’ trait-level mean of belonging predicted within-person decreases in conspiracy belief, *b* = −0.02, 95% CI = [−0.04, < −0.00], β = −0.01, *p* = .029. The corresponding standardized regression coefficient indicates that this is a very small cross-lagged effect. Within-person deviations from participants’ trait-level mean of conspiracy belief did not, however, predict subsequent within-person deviations in belonging, *b* = −0.00, 95% CI = [−0.01, 0.00], β = −0.00, *p* = .181.

#### Meaning in Life

The third model tested whether meaning in life and conspiracy belief were associated longitudinally by estimating an RI-CLPM using data from Times 11–14. The model used data from 55,229 participants (with 112 patterns of missing data). Fit indices for the final RI-CLPM with full stationarity indicate excellent model fit, χ^2^_(17)_ = 749.515, *p* < .001; *CFI* = 0.993, *RMSEA* = 0.028 [0.026, 0.030], *SRMR* = 0.027.

**Figure 3.**
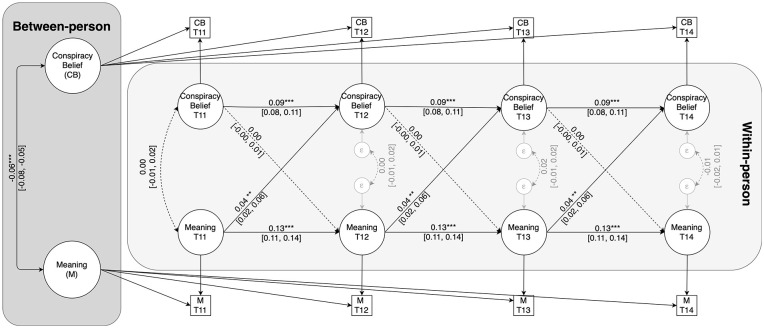
RI-CLPM of the Relationship Between Conspiracy Belief and Meaning in Life. *Note*. Coefficients are unstandardized. Dashed lines represent nonsignificant effects. **p < .01. ***p < .001.

At the between-person level, the random intercepts of belonging and conspiracy belief correlated negatively, *b* = −0.06, 95% CI = [−0.08, −0.05], *p* < .001. Thus, individuals who generally report lower levels of meaning in life also report stronger conspiracy belief across assessments. With respect to the within-person associations, both autoregressive effects were positive and significant, indicating that within-person deviations from participants’ trait-level mean at one wave carried over to within-person deviations in the same variable at the next wave (see Figure 3). Most importantly, within-person deviations from participants’ trait-level meaning in life at one assessment predicted subsequent within-person increases in conspiracy belief, *b* = 0.04, 95% CI = [0.02, 0.06], β = 0.02, *p* = .001. The corresponding standardized estimate indicates that this is a very small cross-lagged effect. Within-person deviations from participants’ trait-level conspiracy belief at one assessment did not, however, predict subsequent deviations in meaning in life, *b* = 0.00, 95% CI = [−0.00, 0.01], β = 0.00, *p* = .438.

#### Self-Esteem

The fourth model tested whether self-esteem and conspiracy belief were associated longitudinally by estimating an RI-CLPM using data from Times 11–14. The model used data from 55,253 respondents (and contained 149 patterns of missing data). Fit indices for the final RI-CLPM with full stationarity indicate excellent model fit, χ^2^_(17)_ = 738.521, *p* < .001; *CFI* = 0.994, *RMSEA* = 0.028 [0.026, 0.029], *SRMR* = 0.026.

**Figure 4.**
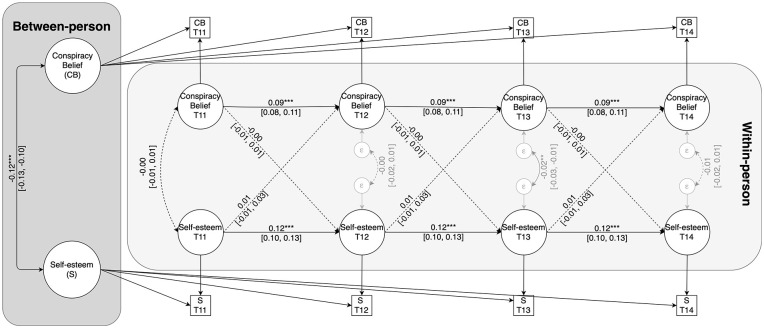
RI-CLPM of the Relationship Between Conspiracy Belief and Self-Esteem. *Note*. Coefficients are unstandardized. Dashed lines represent nonsignificant effects. **p < .01. ***p < .001.

At the between-person level, the random intercepts of belonging and conspiracy belief correlated negatively, *b* = −0.12, 95% CI = [−0.13, −0.10], *p* < .001. Thus, individuals who generally report lower levels of self-esteem also report stronger conspiracy belief across assessments. With respect to within-person associations, both autoregressive effects were positive and significant. As such, within-person deviations from participants’ trait-level mean at one wave carried over to within-person deviations in the same variables at the next wave (see Figure 4). Most importantly, within-person deviations from participants’ trait-level mean in self-esteem at one assessment occasion did not predict subsequent within-person deviations in conspiracy belief, *b* = 0.01, 95% CI = [−0.01, 0.03], β = 0.01, *p* = .351. Similarly, within-person deviations in participants’ trait-level mean of conspiracy belief at one assessment did not predict subsequent deviations in self-esteem, *b* = −0.00, 95% CI = [−0.01, 0.01], β = −0.00, *p* = .866.

## Discussion

Psychological factors have long been thought to play a key role in shaping individuals’ conscious and unconscious motivations to endorse conspiracy theories (Douglas & Sutton, 2023). Unfortunately, empirical evidence to date provides no clear picture of the longitudinal association between psychological need satisfaction and conspiracy belief. Using data from a large, nationwide random sample of New Zealand adults, we established general (between-person) and temporal (within-person) links between conspiracy belief and four psychological needs (control, belonging, self-esteem, and meaning in life) across three-to-four annual waves of data. Consistent with previous studies (Bowes et al., 2023; Liekefett et al., 2023), our results show that people with stronger conspiracy belief generally report lower need satisfaction across all four needs. The strength of the negative association between conspiracy belief and all four needs did, however, vary considerably. For instance, the nonoverlapping 95% CIs for the corresponding point estimates suggest that control is a stronger correlate than meaning in life of between-person differences in conspiracy belief.

Our results also provide critical insights into the longitudinal within-person associations that emerge over time. Somewhat unexpectedly, these within-person associations varied across psychological needs. For example, although the lagged associations both control and belonging had with conspiracy belief were negative (as expected), meaning in life had a positive lagged association with conspiracy belief. These associations were, however, rather modest in size. Indeed, the effect sizes of the significant cross-lagged associations ranged from very small to small. Nevertheless, these results suggest that both *lower* and *higher* need satisfaction can predict increases in conspiracy belief over time.

### Lower Need Satisfaction May Contribute to Conspiracy Belief

Consistent with the argument that conspiracy belief stems from a lack of psychological need satisfaction (Douglas et al., 2017; Poon et al., 2020), lower-than-usual levels of control and belonging preceded increases in conspiracy belief a year later. These results corroborate and extend the extant literature showing that experimental threats to basic needs can strengthen conspiracy belief (Poon et al., 2020). That the effect was noticeably larger for control than for belonging suggests that fluctuations in a person’s feeling of control better predict conspiracy belief than do fluctuations in their sense of belonging. This pattern also emerges with the between-person effects, suggesting that control is a more relevant predictor than sense of belonging. Although we must reiterate that these within-person cross-lagged effects are small, their presence provides important theoretical insights. Indeed, these cross-lagged effects demonstrate that even subtle variations in control and belonging are associated with conspiracy belief 1 year later, underscoring the sensitivity of belief systems to underlying psychological states. Nevertheless, other relevant factors likely affect individuals’ conspiracy belief. Indeed, Stojanov and Halberstadt’s (2020) recent meta-analysis reveals a more complex association between control experiences and conspiracy belief, as domain-specific (compared with general) conspiracy belief appears to be more easily impacted by a loss of control (see also Stojanov et al., 2021). In this study, within-person fluctuations in perceived control even preceded stronger *general* conspiracy belief. As such, the importance of real-life (compared with experimentally manipulated) changes in perceived control may be a promising area of future research (see also Bukowski et al., 2024).

Given that conspiracy belief correlates with stigmatization and expected social exclusion (Lantian et al., 2018), seeking refuge in conspiracy theories to cope with low levels of belonging or control can be risky. Indeed, both theoretical and empirical work suggests that conspiracy belief is, if anything, associated with lower levels of belonging and control (Douglas et al., 2017; Liekefett et al., 2023). Similarly, other strands of research reveal that some ways of restoring control can backfire (e.g., less personal control is associated with narcissistic in-group positivity; Cichocka et al., 2018). As such, people may rely on conspiracy belief to restore control and belonging only if there are no alternative, less stigmatizing, and possibly more efficient, coping strategies (e.g., Marchlewska et al., 2022).

It is important to note that the data analyzed here were collected during different stages of the COVID-19 pandemic (except for early responders at Time 11 and all of Time 14 data). Given that societal crises can increase the prevalence of conspiracy theories (van Prooijen & Douglas, 2017), the ongoing pandemic may have fueled conspiracy belief during our data collection period. Interestingly, despite the crisis, New Zealanders reported high trust in politicians and satisfaction with the government’s performance in the early phases of the global crisis (Sibley et al., 2020). Nevertheless, the timing of data collection may have impacted participants’ interpretations of our measures. For example, participants may have interpreted the perceived control items in terms of personal aspects of their lives before the emergence of COVID-19 (e.g., control over who to be with), but could have shifted to political aspects of control during the pandemic (e.g., control over current pandemic-related restrictions). Still, given that measurement invariance tests did not indicate changes in the factor structure for our three-item constructs, this alternative explanation appears less plausible. Nevertheless, future work using three-item (or more) measures of control is needed to examine this possibility.

Notably, belonging had weaker temporal associations with conspiracy belief compared with control. Indeed, belonging appears to function better as a cross-sectional (vs. longitudinal) predictor of conspiracy belief. Specifically, control and belonging had the strongest negative between-person associations with conspiracy belief. Consistent with meta-analytic results for control (Stojanov & Halberstadt, 2020), these results highlight the strong predictive, but not necessarily causal, function of these needs for conspiracist thoughts.

### Greater Need Satisfaction May Contribute to Conspiracy Beliefs

Only one stable within-person cross-lagged path emerged with respect to conspiracy belief and meaning in life: Individuals who experienced a temporary increase from their trait-level meaning in life reported higher-than-usual levels of conspiracy belief a year later. Similarly, the cross-lagged path from self-esteem to conspiracy belief was positive, but the effect was not significant. These results indicate that *higher* psychological need satisfaction may increase conspiracy belief (within-person). That said, people who report *higher* meaning in life (as well as higher self-esteem) also generally report *lower* conspiracy belief (between-person). These cross-lagged results are surprising given that perceiving one’s life as meaningful is often associated with positive outcomes (Steger et al., 2006). It thus raises the question of *why* individuals who experience a temporary increase in their meaning in life also experience a subsequent increase in their belief that authorities often hide the truth about major world events.

Some previous research reveals a positive relationship between conspiracy belief and the presence of, and search for, meaning in life (Schöpfer et al., 2023), as well as self-esteem (Alsuhibani et al., 2022; Cichocka et al., 2016). Interestingly, Schöpfer et al. (2023) argued that conspiracy belief may offer people a purpose by giving them an opportunity to become active, make a difference, and have an impact on the world (e.g., by pursuing collective action). However, the present data provide no evidence that increased conspiracy belief increases meaning in life. Rather, a temporary increase in meaning in life contributes to stronger conspiracy belief. Similarly, Cichocka et al. (2016) found that conspiracy belief is linked to narcissism rather than high self-esteem. These results may explain why we failed to find a within-person longitudinal relationship between self-esteem and conspiracy belief.

Alternatively, threats to meaning in life may trigger a *search for*, but not necessarily a *loss in*, meaning (Graeupner & Coman, 2017). Similarly, the pursuit of self-esteem may not be exclusively due to feelings of low self-worth, but rather, may be determined by multiple factors (e.g., narcissism; Horvath & Morf, 2009). Thus, the types of threats to perceived meaning or self-esteem that foster conspiracy belief may not be captured by our measures. Experimental manipulations of perceived meaning in life are, therefore, needed to increase understanding of the nexus between meaning in life and conspiracy belief.

### Limitations and Future Directions

This study utilized an existing data set that includes a general question about conspiracy belief. Although our results provide critical insights that increase understanding of the needs that underlie conspiracy belief, there may be important differences related to endorsement of more specific conspiracy theories (e.g., belief in COVID-19-related conspiracy theories). Specifically, temporal associations between psychological needs and more domain-specific conspiracy theories may differ from the results reported here, given that “general” and “specific” conspiracy beliefs differ in important ways (Sutton & Douglas, 2020). In addition, our measure of conspiracy belief relied on a modified single-item measure (Lantian et al., 2016). As such, it may be less valid than other scales using multiple items and/or focusing on conspiracy theories that originate from government agencies. Nevertheless, the large and longitudinal nature of our data set offers valuable insights into the association between conspiracy belief and psychological needs. Future research may expand on these results, for instance, by using multi-item scales (which runs the risk of increasing participant fatigue).

It is also important to remember that our three-item measure of belongingness had low reliability at all four assessments. That said, low levels of reliability tend to attenuate the relationship between variables (Crocker & Algina, 1986). As such, the cross-lagged effects of belonging on conspiracy belief may be underestimated in this study. Accordingly, future studies may wish to use longer multi-item scales to examine this possibility.

There are also three main points to consider with respect to our analyses. First, the temporal lag between assessments may affect the strength of associations detected here (Liekefett et al., 2023). For instance, psychological needs may have stronger within-person effects on conspiracy belief when examining time spans shorter than 1 year. Second, because most of our variables were assessed by scales with only one or two items, our analyses included indices (i.e., means) instead of latent variables (which require three or more items). And due to convergence issues when attempting to run multiple-indicator RI-CLPMs, we also modeled our two three-item measures (i.e., self-esteem and felt belonging) as single-item indices. Consequently, our models do not adjust for measurement error. As such, future research should consider using long-form measures and estimating a multiple-indicator RI-CLPM (Mulder & Hamaker, 2021). Third, although longitudinal data allow one to investigate the temporal associations between conspiracy belief and psychological needs, they do not provide a definitive test of causality. Nevertheless, investigating these temporal associations offers a first step into understanding the psychological antecedents and consequences of conspiracy belief.

Finally, although our use of an RI-CLPM allows us to adjust for between-person effects while focusing on within-person deviations from participants’ trait-level means (Hamaker et al., 2015), the approach is not without its limitations. For one, because the focus is on time-specific departures from trait-level means, some argue that RI-CLPMs are better-suited for testing short-term processes and that the approach overlooks important changes between individuals (Hamaker, 2023; Lüdtke & Robitzsch, 2022). Others note that panel models in general (i.e., both the traditional CLPM and RI-CLPM) can yield biased estimates if the phenomenon being studied is nonstationary and/or has yet to reach a state of equilibrium (i.e., the means, variances, and covariances are still changing over time; see Andersen, 2022). Accordingly, alternative approaches to measuring within-person change have been proposed including growth curve models (e.g., Grimm et al., 2017), latent curve models with structured residuals (Curran et al., 2014), the general cross-lagged panel model (Zyphur et al., 2020), and mixed-effects location scale models (Wright & Jackson, 2025)—to name but just a few. Generally, each of these approaches has its own unique strengths and weaknesses (Osborne & Little, 2024). For instance, growth curve models are flexible analytic tools that help identify average rates of change in a given construct over time and allow for the lags between assessments to vary between participants (Grimm et al., 2017). In contrast, an RI-CLPM requires the lags between assessments to be similar across the sample population. Yet RI-CLPMs can assess potential reciprocal effects within the same model and are increasingly becoming the method of choice for studies seeking to identify the temporal ordering of within-person change (see Osborne & Little, 2024). Thus, the RI-CLPM is particularly well-suited to address our research question about the within-person temporal association between need satisfaction and conspiracy belief.

## Conclusion

Although psychological factors are thought to impact the motivation to believe in conspiracy theories, current literature does not provide a comprehensive picture of the longitudinal associations between psychological needs and conspiracy belief. Using four annual waves of a large-scale survey from New Zealand, we identify important stable between-person and temporal within-person associations between four commonly investigated needs (namely control, belonging, self-esteem, and meaning in life) and conspiracy belief. Consistent with existing research, our results demonstrate that individuals with unmet needs report stronger conspiracy belief. Within-person analyses, however, reveal that both lower (i.e., control, belonging) and higher (i.e., meaning in life) need satisfaction can precede increases in conspiracy belief. Together, these results provide novel insights into the psychological factors that foster conspiracy belief and suggest that the frustration of psychological needs does not always lead to conspiracy belief.

## Notes

1. Due to the low reliability, we also created an index for belonging without the third item and estimated an RI-CLPM including this index and conspiracy across all four time points. We report the results of this model here: https://osf.io/h9zfq/.
